# WISP-3 promotes PDGF-B-mediated angiogenesis through suppression of let-7b-5p in rheumatoid arthritis

**DOI:** 10.3389/fimmu.2026.1842824

**Published:** 2026-07-29

**Authors:** Guo-Shou Wang, Sheng-Mou Hou, Syuan-Ling Lin, Shan-Chi Liu, Chao-Ju Chen, Chih-Hsin Tang, Chih-Yang Lin

**Affiliations:** 1 Department of Medicine, MacKay Medical University, New Taipei City, Taiwan; 2 Department of Orthopedic Surgery, MacKay Memorial Hospital, Taipei, Taiwan; 3 Department of Research, Taiwan Blood Services Foundation, Taipei, Taiwan; 4 The Director’s Office, Shin Kong Wu Ho-Su Memorial Hospital, Taipei, Taiwan; 5 Translational Medicine Research Center, China Medical University Hospital, Taichung, Taiwan; 6 Graduate Institute of Biomedical Sciences, China Medical University, Taichung, Taiwan; 7 Institute of Biomedical Sciences, Mackay Medical University, New Taipei City, Taiwan; 8 Translational Medicine Center, Shin-Kong Wu Ho-Su Memorial Hospital, Taipei, Taiwan; 9 Department of Pharmacology, School of Medicine, China Medical University, Taichung, Taiwan; 10 Department of Medical Laboratory Science and Biotechnology, Asia University, Taichung, Taiwan; 11 Chinese Medicine Research Center, China Medical University, Taichung, Taiwan; 12 Department of Medical Research, China Medical University Hospital, China Medical University, Taichung, Taiwan

**Keywords:** angiogenesis, hsa-let-7b-5p, PDGF-B, rheumatoid arthritis, WISP-3

## Abstract

Rheumatoid arthritis (RA) is characterized by chronic synovial inflammation accompanied by pathological angiogenesis, which contributes to pannus formation and disease progression. However, the upstream mechanisms regulating pathological angiogenesis in RA remain incompletely understood. In this study, bioinformatic analysis of two independent Gene Expression Omnibus (GEO) datasets, immunohistochemical analysis of human synovial tissues, and functional studies using MH7A synovial fibroblasts and HUVEC tube formation assays were performed to investigate the role of WNT1-inducible signaling pathway protein 3 (WISP-3) in RA angiogenesis. WISP-3 expression was consistently elevated in RA synovial tissues compared with healthy controls. Recombinant WISP-3 significantly enhanced endothelial tube formation and increased platelet-derived growth factor-B (PDGF-B) expression in MH7A cells. Mechanistically, WISP-3 activated the PI3K/p85/Akt/mTOR signaling pathway, whereas pharmacological inhibition or siRNA-mediated knockdown of p85, Akt, or mTOR markedly attenuated PDGF-B expression and angiogenic activity. WISP-3 also suppressed hsa-let-7b-5p expression, and restoration of hsa-let-7b-5p using a synthetic mimic significantly reduced WISP-3-induced PDGF-B expression and endothelial tube formation. Luciferase reporter assays further confirmed PDGF-B as a direct target of hsa-let-7b-5p. These findings identify a previously unrecognized WISP-3/p85/Akt/mTOR/hsa-let-7b-5p/PDGF-B signaling axis that promotes pathological angiogenesis and provides new mechanistic insight into synovial vascular remodeling in RA.

## Introduction

Rheumatoid arthritis (RA) is a chronic systemic autoimmune disease characterized by persistent synovial inflammation, fibroblast-like synoviocyte hyperplasia, and progressive destruction of cartilage and bone (1). In addition to chronic inflammation, pathological angiogenesis is a hallmark of RA and plays a central role in disease progression (2, 3). The expansion of the synovial microvasculature not only supplies oxygen and nutrients to the proliferating pannus but also facilitates the continuous recruitment of inflammatory cells, including macrophages, T lymphocytes, and neutrophils, thereby perpetuating synovial inflammation and joint destruction (2, 4). Because angiogenesis contributes to both vascular remodeling and immune cell infiltration, identifying the molecular mechanisms that regulate this process may provide new therapeutic opportunities for RA.

Among the angiogenic mediators implicated in RA, platelet-derived growth factor-B (PDGF-B) has emerged as an important regulator of synovial vascular remodeling (5). In addition to its well-established role in mesenchymal cell proliferation, PDGF-B promotes endothelial cell activation, vascular maturation, and tissue remodeling under pathological conditions (6). Elevated PDGF-B expression has been detected in RA synovial tissues, where it is co-expressed with fibroblast growth factor-1 and other angiogenic mediators (7). More recently, high-mobility group box-1 was shown to enhance PDGF-B production and promote pathological angiogenesis in RA, further supporting a pivotal role for PDGF-B in synovial vascular expansion and disease progression (5). Despite these observations, the upstream molecular mechanisms responsible for PDGF-B dysregulation in RA remain largely undefined.

Accumulating evidence indicates that microRNAs (miRNAs) are critical post-transcriptional regulators of gene expression and contribute substantially to RA pathogenesis by modulating synoviocyte activation, inflammatory signaling, osteoclastogenesis, and angiogenesis (8–10). Dysregulated miRNAs have been shown to influence multiple aspects of RA progression (11, 12). For example, miR-378 promotes angiogenesis and osteoclastogenesis through enhancement of endoplasmic reticulum stress (13). Moreover, recent profiling studies have demonstrated that several members of the let-7 family are differentially expressed in patients with RA and are closely associated with immune regulation and disease progression, highlighting the importance of this miRNA family in RA pathogenesis (14). Despite these advances, the specific miRNAs linking upstream signaling molecules to angiogenic mediators such as PDGF-B remain largely unknown. Identifying these regulatory miRNAs may therefore provide new mechanistic insights into pathological angiogenesis in RA.

Members of the CCN family of matricellular proteins have emerged as important regulators of tissue remodeling, inflammation, and angiogenesis in musculoskeletal diseases (15). WNT1-inducible signaling pathway protein-3 (WISP-3, also known as CCN6) has been reported to regulate cell proliferation, migration and angiogenic factor production in several pathological conditions (16). In particular, WISP-3 promotes VEGF-A-dependent angiogenesis in chondrosarcoma cells (17). More recently, WISP-3 was found to stimulate VEGF-C-dependent lymphangiogenesis via miRNA suppression in chondrosarcoma (18). These findings suggest that WISP-3 can regulate vascular remodeling through growth factor- and miRNA-dependent mechanisms. Nevertheless, whether WISP-3 contributes to pathological angiogenesis in RA and whether it regulates PDGF-B expression remain unknown.

In the present study, we investigated the role of WISP-3 in RA-associated angiogenesis and elucidated the underlying molecular mechanisms. We demonstrate that WISP-3 promotes PDGF-B expression through activation of the p85/Akt/mTOR signaling pathway and suppression of hsa-let-7b-5p, leading to enhanced angiogenic activity. Our findings identify a previously unrecognized WISP-3–hsa-let-7b-5p–PDGF-B signaling axis that contributes to pathological angiogenesis in RA and may represent a potential therapeutic target for limiting synovial vascular remodeling.

## Materials and methods

### Materials

The following antibodies were used for Western blot analysis: phospho-p85 (Tyr458)/p55 (Tyr199) (#4228), phospho-Akt (Ser473) (D9E) (#4060), total Akt (C67E7) (#4691), phospho-mTOR (Ser2448) (D9C2) (#5536), and total mTOR (7C10) (#2983) were obtained from Cell Signaling Technology (Danvers, MA, USA). The antibody against p85α (HL1398; #GTX636838) was purchased from GeneTex (Hsinchu, Taiwan). Anti–PDGF-B (Cat. No. AF5179) was purchased from Affinity Biosciences (Jiangsu, China), *and anti-WISP-3 (Cat. No. PA5-98241) was purchased from Invitrogen (Carlsbad, CA, USA).* β-actin (#A5441*; Sigma-Aldrich, St. Louis, MO, USA*) was used as a loading control. Small interfering RNAs (siRNAs) targeting p85, Akt, and mTOR, *together with a* non-targeting control siRNA, were purchased from Santa Cruz Biotechnology (Santa Cruz, CA, USA). Recombinant human WISP-3 was obtained from PeproTech (Rocky Hill, NJ, USA). Dulbecco*’*s Modified Eagle Medium (DMEM) and fetal bovine serum (FBS) were purchased from Gibco (Grand Island, NY, USA). All other reagents were obtained from Sigma-Aldrich unless otherwise stated.

### Bioinformatics analysis of clinical datasets

Gene expression profiles of CCN family members were analyzed using the GSE77298 and GSE12021 datasets retrieved from the Gene Expression Omnibus (GEO) database. The GSE77298 dataset includes synovial tissue samples from healthy controls (n = 7) and patients with RA (n = 16). The GSE12021 dataset comprises synovial tissues from normal controls (n = 4), osteoarthritis (OA) patients (n = 10), and RA patients (n = 9). The datasets were processed and analyzed as provided in GEO without additional normalization.

### Human synovial tissue specimens and immunohistochemistry

Human synovial tissues were collected from 6 patients with end-stage RA and 9 trauma controls at China Medical University Hospital after written informed consent. The study was approved by the Institutional Review Board (CMUH109-REC2-181). Sections were stained with anti-WISP-3 (1:250) or anti-PDGF-B (1:250). Signals were visualized using the NovoLink Polymer Detection System with DAB and counterstained with hematoxylin. Staining intensity was semi-quantitatively scored on a 1–5 scale by two independent blinded investigators.

### Cell lines and culture

The human rheumatoid arthritis synovial fibroblast cell line MH7A was obtained from RIKEN (Ibaraki, Japan). Cells were maintained in DMEM supplemented with 10% FBS and antibiotics (100 U/mL penicillin and 100 μg/mL streptomycin) at 37 °C in a humidified incubator with 5% CO_2_.

Human umbilical vein endothelial cells (HUVECs; BCRC No. H-UV001) were obtained from the Bioresource Collection and Research Center (Hsinchu, Taiwan) and cultured under the same conditions (19).

### Cell counting kit-8 assay

Cell viability was assessed using a CCK-8 (Dojindo Molecular Technologies, Kumamoto, Japan). MH7A cells were seeded into 96-well plates at a density of 5 × 10^3^ cells per well and allowed to adhere overnight. Cells were then treated with recombinant human WISP-3 at the indicated concentrations (0, 10, 30, and 100 ng/mL) for 24 h. Subsequently, 10 μL of CCK-8 reagent was added to each well and incubated at 37 °C for 1 h. The absorbance at 450 nm was measured using a microplate reader, and cell viability was expressed as the percentage of the untreated control.

### Western blot analysis

MH7A cells were pretreated with the indicated inhibitors for 30 min or transfected with the indicated siRNAs for 24 h prior to stimulation with recombinant human WISP-3 (100 ng/mL). For phosphorylation studies, cells were harvested at 0, 10, 15, 30, 60, and 120 min after WISP-3 stimulation. For analysis of PDGF-B protein expression, cells were stimulated with WISP-3 for 24 h. Cell lysates were prepared as previously described (20, 21). Equal amounts of protein were separated by SDS-PAGE and transferred onto PVDF membranes. After blocking with 4% non-fat milk in TBST for 1 h at room temperature, the membranes were incubated with primary antibodies against phospho-p85, p85, phospho-Akt, Akt, phospho-mTOR, mTOR, PDGF-B, or β-actin either at room temperature for 1 h or at 4 °C overnight. After washing with TBST, membranes were incubated with HRP-conjugated secondary antibodies for 1 h at room temperature. Protein signals were detected using an enhanced chemiluminescence reagent and visualized with a Fujifilm LAS-3000 imaging system. Band intensities were quantified using UN-SCAN-IT gel software and normalized to β-actin.

### Quantitative real-time polymerase chain reaction

MH7A cells were pretreated with the indicated inhibitors for 30 min or transfected with the indicated siRNAs or hsa-let-7b-5p mimic for 24 h prior to stimulation with recombinant human WISP-3 (100 ng/mL) for 24 h. Total RNA was extracted using TRIzol reagent (MDBio, Taipei, Taiwan). RNA concentration and purity were determined by measuring the A260/A280 ratio using a NanoDrop spectrophotometer. First-strand cDNA was synthesized using a reverse transcription kit (Invitrogen, Carlsbad, CA, USA). qRT-PCR was performed using SYBR Green Master Mix in a final reaction volume of 20 μL. Relative mRNA expression levels were calculated using the 2^−^ΔΔCt method and normalized to GAPDH (20). Relative hsa-let-7b-5p expression was normalized to U6 small nuclear RNA. Primer sequences are listed in **Supplementary Table 1**.

### siRNA and miRNA transfection

Small interfering RNAs (siRNAs) targeting p85, Akt, and mTOR, together with a non-targeting control siRNA, were purchased from Santa Cruz Biotechnology (Santa Cruz, CA, USA). Synthetic hsa-let-7b-5p mimic and the corresponding negative control mimic were obtained from AllBio RNA System (AllBio, Taichung, Taiwan). MH7A cells were transfected with siRNAs or miRNA mimics at a final concentration of 50 nM using Lipofectamine RNAiMAX (Invitrogen, Carlsbad, CA, USA) according to the manufacturer’s instructions. Briefly, siRNAs or miRNA mimics and Lipofectamine RNAiMAX were separately diluted in Opti-MEM^®^ Reduced Serum Medium (Gibco, Grand Island, NY, USA) and incubated for 5 min at room temperature. The diluted reagents were then combined and incubated for an additional 20 min to allow formation of transfection complexes. The complexes were added to MH7A cells and incubated overnight. Twenty-four hours after transfection, the cells were stimulated with recombinant human WISP-3 (100 ng/mL) for an additional 24 h before subsequent analyses.

### Dual-luciferase reporter assay

The wild-type (WT) or mutant (MUT) PDGF-B 3′-untranslated region (3′UTR) luciferase reporter plasmids containing the predicted hsa-let-7b-5p binding site were synthesized by MDBio Inc. (Taipei, Taiwan). MH7A cells were co-transfected with the WT or MUT reporter plasmids together with either the hsa-let-7b-5p mimic or the corresponding negative control mimic using Lipofectamine 2000 (Thermo Fisher Scientific, Waltham, MA, USA). Twenty-four hours after transfection, luciferase activities were measured using the Dual-Luciferase^®^ Reporter Assay System (Promega, Madison, WI, USA) according to the manufacturer’s instructions. Firefly luciferase activity was normalized to Renilla luciferase activity, and the relative luciferase activity was expressed as the fold change relative to the corresponding control group.

### Preparation of conditioned medium (CM) and HUVEC tube formation assay

MH7A cells were pretreated with the indicated pharmacological inhibitors for 30 min or transfected with the indicated siRNAs or hsa-let-7b-5p mimic for 24 h prior to stimulation with recombinant human WISP-3 (100 ng/mL) for an additional 24 h. The CM was collected, centrifuged at 3,000 × g for 10 min to remove cellular debris, and stored at -80 °C until use. For PDGF-B neutralization experiments, CM was incubated with either a PDGF-B-neutralizing antibody or control IgG for 1 h at room temperature before use. To evaluate the direct effect of extracellular WISP-3, recombinant human WISP-3 (100 ng/mL) was added directly to CM collected from untreated MH7A cells immediately before the tube formation assay.

For the tube formation assay, Matrigel (120 μL/well; Corning, Corning, NY, USA) was added to 48-well plates and allowed to polymerize at 37 °C for 30 min. HUVECs were seeded at a density of 3 × 10^5^ cells per well in a 1:1 mixture of endothelial growth medium and CM. After incubation for 6 h at 37 °C, tube-like structures were photographed under a light microscope. Angiogenic activity was quantified by counting the number of branch points in five randomly selected fields using ImageJ software.

### Statistical analysis

All experiments were performed at least three times independently. Data are presented as mean ± standard deviation (SD). Differences between two groups were analyzed using an unpaired two-tailed Student’s t-test. Comparisons among three or more groups were analyzed using one-way analysis of variance (ANOVA) followed by Tukey’s *post hoc* test. Statistical analyses were performed using GraphPad Prism 8.0, and p < 0.05 was considered statistically significant.

## Results

### Elevated WISP-3 expression is associated with RA synovial tissues

**Figure 1:**
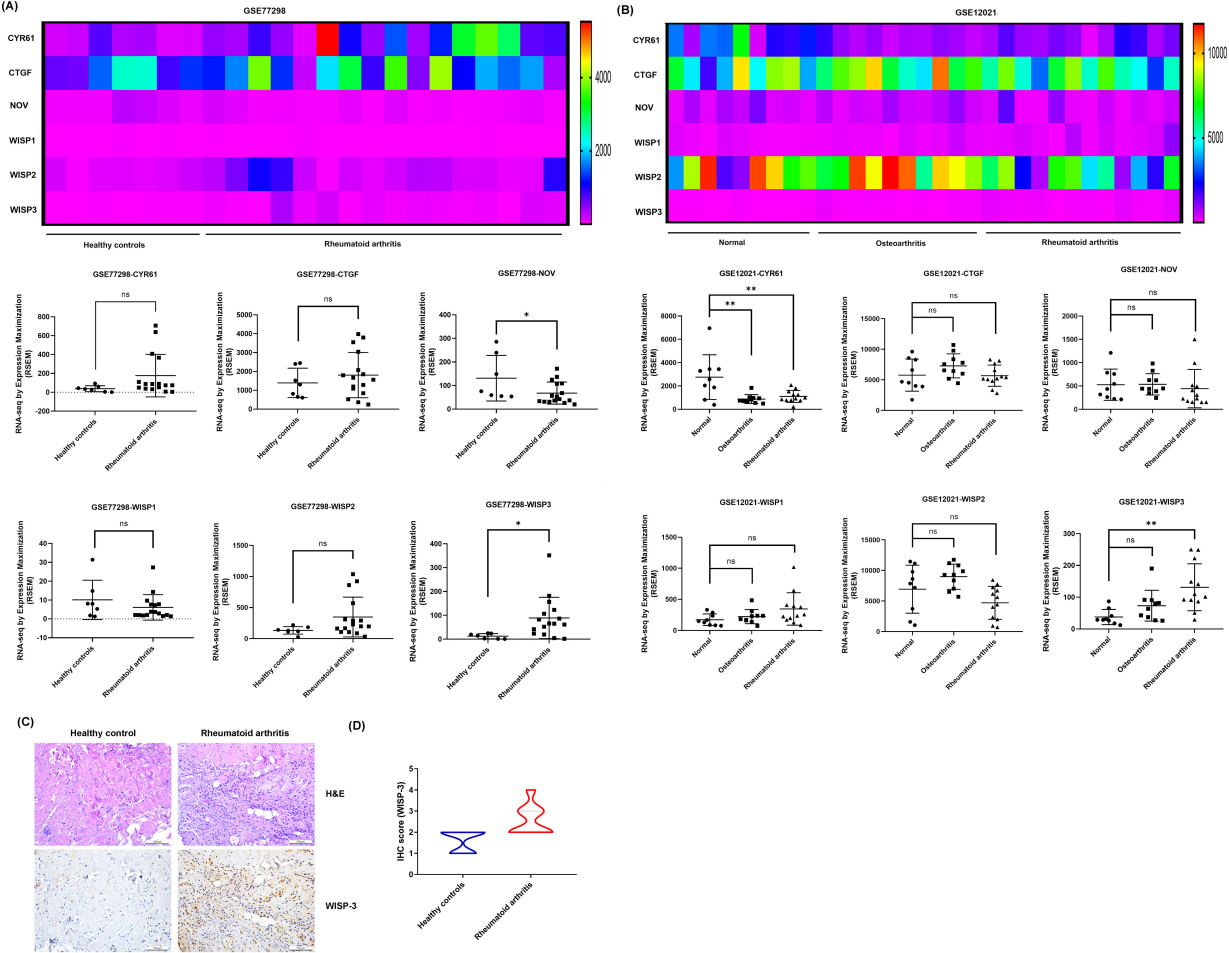
WISP-3 expression is elevated in RA synovial tissues. **(A)** Heatmap and quantification analysis of CCN family gene expression in healthy controls (n = 7) and RA synovial tissues (n = 16) from the GSE77298 dataset. **(B)** Heatmap and quantitative analysis of CCN family gene expression in normal synovial tissues (n = 4), OA synovial tissues (n = 10), and RA synovial tissues (n = 9) from the (GSE12021) dataset. **(C)** Representative H&E staining and IHC staining for WISP-3 in healthy control and RA synovial tissues. **(D)** Semi-quantitative analysis of WISP-3 IHC staining scores. Data are presented as mean ± SD from at least three independent experiments. Statistical significance was analyzed using an unpaired Student’s t-test for two-group comparisons and one-way ANOVA followed by Tukey’s *post hoc* test for multi-group comparisons. *p < 0.05, **p < 0.01, ***p < 0.001 compared with the control group; ns, not significant.

Analysis of two independent GEO datasets revealed that WISP-3 was consistently upregulated in RA synovial tissues. In the GSE77298 dataset, which included healthy controls (n = 7) and RA synovial tissues (n = 16), WISP-3 expression was significantly higher in RA samples than in healthy controls, whereas other CCN family members exhibited variable expression patterns (**Figure 1A**). Similar findings were observed from the GSE12021 dataset, included normal synovial tissues (n = 4), osteoarthritis (OA) tissues (n = 10), and RA synovial tissues (n = 9), in which WISP-3 expression was significantly elevated in RA tissues compared with normal controls, while the expression profiles of other CCN family members remained less consistent (**Figure 1B**). To validate these transcriptomic observations at the protein level, WISP-3 expression was further examined by immunohistochemistry. WISP-3 immunoreactivity was markedly stronger in RA synovial tissues than in healthy controls (**Figure 1C**), and semi-quantitative analysis confirmed significantly increased WISP-3 protein expression in RA specimens (**Figure 1D**). Together, these findings demonstrate that WISP-3 is consistently upregulated in RA synovial tissues at both the mRNA and protein levels.

### WISP-3 promotes angiogenic activity through PDGF-B upregulation in MH7A cells

**Figure 2:**
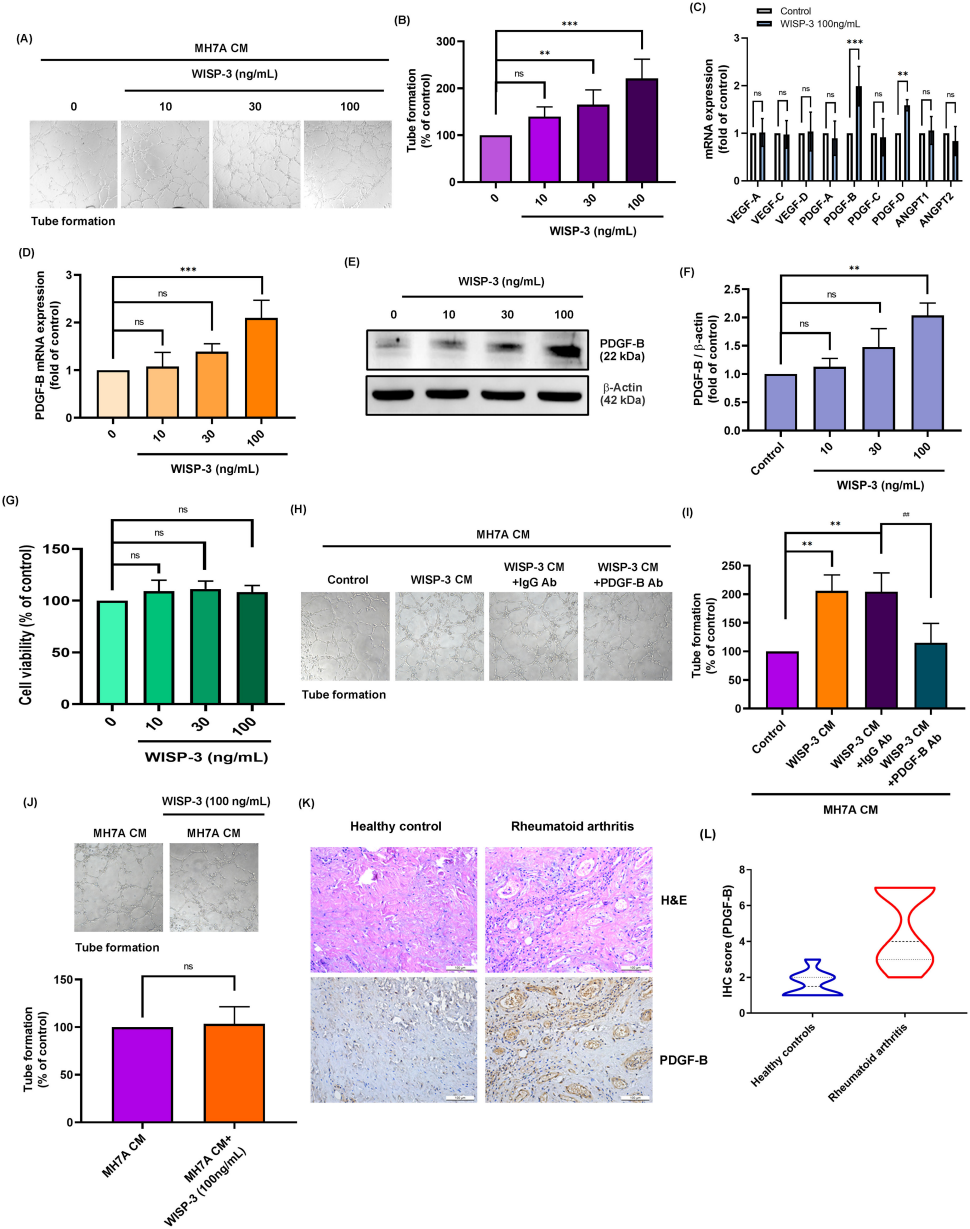
WISP-3 promotes angiogenic activity through PDGF-B upregulation in MH7A cells. **(A, B)** Representative images and quantification of HUVEC tube formation following incubation with CM collected from MH7A cells treated with the indicated concentrations of WISP-3 (0-100 ng/mL). **(C)** mRNA expression of angiogenesis-related genes in MH7A cells following WISP-3 stimulation. **(D)** PDGF-B mRNA expression in MH7A cells treated with increasing concentrations of WISP-3. **(E, F)** Representative western blot and densitometric analysis of PDGF-B protein expression. **(G)** Cell viability of MH7A cells following WISP-3 treatment. **(H, I)** Representative images and quantitative analysis of HUVEC tube formation following incubation with CM treated with control IgG or a PDGF-B-neutralizing antibody. **(J)** Representative images and quantitative analysis of HUVEC tube formation following supplementation of recombinant WISP-3 (100 ng/mL) to conditioned medium collected from MH7A cells. **(K, L)** Representative H&E staining, IHC staining for PDGF-B, and semi-quantitative analysis of PDGF-B expression in synovial tissues from healthy controls and patients with rheumatoid arthritis. Data are presented as the mean ± SD from at least three independent experiments. Statistical significance was analyzed using one-way ANOVA followed by Tukey’s *post hoc* test. *p < 0.05, **p < 0.01, ***p < 0.001 versus the control group; #*p* < 0.05, ##*p* < 0.01, ###*p* < 0.001 versus the WISP-3-treated group; ns, not significant.

To investigate whether WISP-3 contributes to angiogenesis in RA. MH7A cells were stimulated with 10, 30, and 100 ng/mL recombinant WISP-3, and CM was subsequently collected and applied to HUVECs. CM derived from WISP-3-treated MH7A cells significantly enhanced endothelial tube formation in a dose-dependent manner (**Figures 2A, B**). To identify the angiogenic mediator responsible for this effect, we examined the expression of several candidate angiogenic factors. Among the angiogenic factors examined, PDGF-B showed the most prominently induction following WISP-3 stimulation and was therefore selected for subsequent mechanistic investigation (**Figure 2C**). Consistent with these findings, WISP-3 increased PDGF-B expression at both the mRNA and protein levels in a concentration-dependent manner (**Figures 2D–F**), without affecting MH7A cell viability (**Figure 2G**). These results indicate that WISP-3 promotes angiogenic activity, at least in part, through upregulation of PDGF-B. To determine whether PDGF-B mediates the pro-angiogenic activity of WISP-3, CM from WISP-3-treated MH7A cells was incubated with a PDGF-B-neutralizing antibody before being applied to HUVECs. Neutralization of PDGF-B markedly attenuated WISP-3-induced endothelial tube formation, whereas control IgG had no detectable effect (**Figures 2H, I**), indicating that PDGF-B is a major mediator of the angiogenic activity induced by WISP-3.

To exclude the possibility that recombinant WISP-3 directly stimulates endothelial cells, recombinant WISP-3 (100 ng/mL) was added to conditioned medium collected from untreated MH7A cells before incubation with HUVECs. As shown in **Figure 2J**, direct supplementation with recombinant WISP-3 did not significantly affect endothelial tube formation. These findings indicate that recombinant WISP-3 alone does not directly promote angiogenesis in HUVECs and further support that the enhanced tube formation induced by conditioned medium from WISP-3-stimulated MH7A cells is primarily attributable to PDGF-B released by MH7A cells rather than to residual recombinant WISP-3. To further assess the clinical relevance of these findings, PDGF-B expression was examined in synovial tissues obtained from patients with RA and healthy controls by immunohistochemistry. PDGF-B immunoreactivity was markedly increased in RA synovial tissues compared with healthy controls, and semi-quantitative analysis confirmed significantly higher PDGF-B expression in the RA group (**Figures 2K, L**). Together, these findings identify PDGF-B as a clinically relevant downstream effector of WISP-3 and support its contribution to pathological angiogenesis in rheumatoid arthritis.

### p85 signaling mediates WISP-3-induced PDGF-B expression and angiogenic activity in MH7A cells

**Figure 3:**
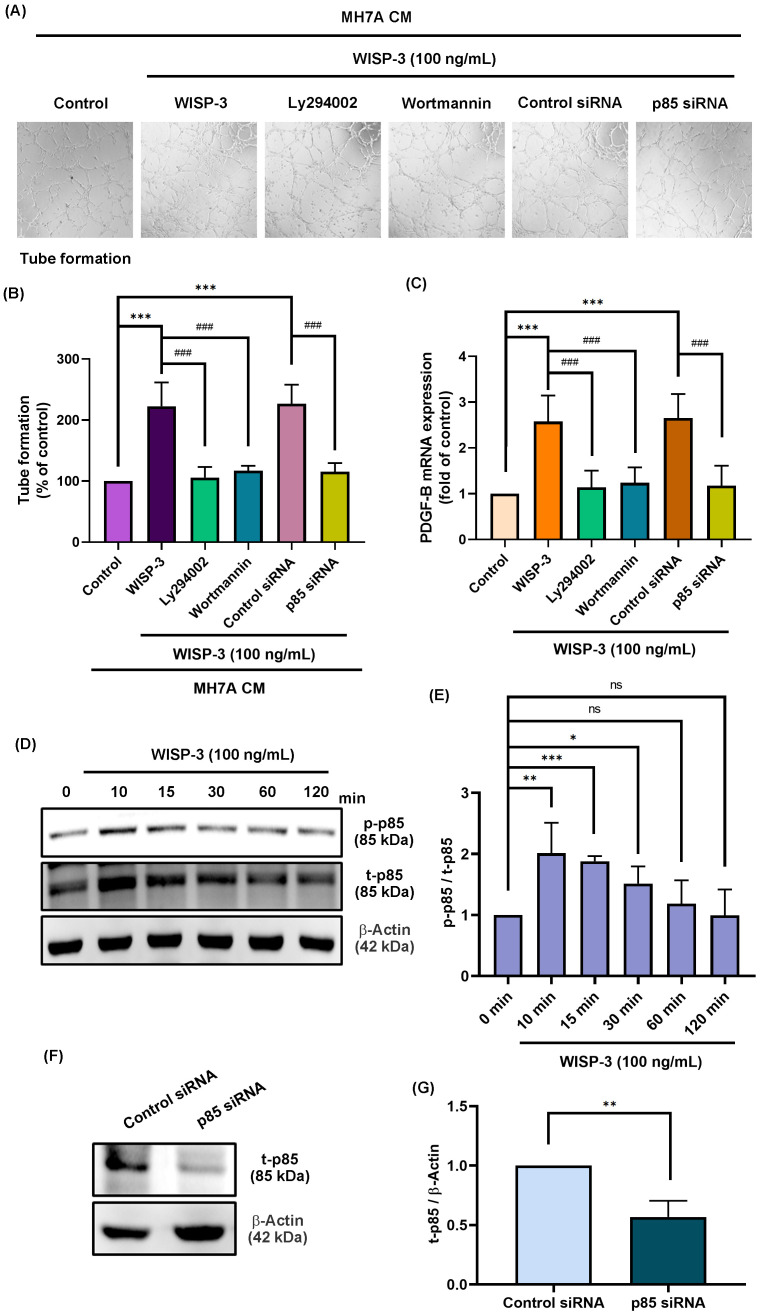
p85 signaling mediates WISP-3-induced PDGF-B expression and angiogenesis. **(A, B)** Representative images and quantitative analysis of HUVEC tube formation using CM collected from MH7A cells pretreated with LY294002 (10 μM), Wortmannin (5 μM), or transfected with p85 siRNA prior to stimulation with recombinant WISP-3 (100 ng/mL). **(C)** PDGF-B mRNA expression in MH7A cells pretreated with LY294002 (10 μM), Wortmannin (5 μM), or transfected with p85 siRNA prior to stimulation with recombinant WISP-3 (100 ng/mL). **(D, E)** Representative Western blot and quantitative analysis of p85 phosphorylation following stimulation with recombinant WISP-3 (100 ng/mL) for the indicated time periods. **(F, G)** Representative Western blot and quantitative analysis confirming p85 knockdown efficiency. Data are presented as the mean ± SD from at least three independent experiments. Statistical significance was analyzed using one-way ANOVA followed by Tukey’s *post hoc* test. *p < 0.05, **p < 0.01, ***p < 0.001 versus the control group; #*p* < 0.05, ##*p* < 0.01, ###*p* < 0.001 versus the WISP-3-treated group; ns, not significant.

Because PI3K signaling has been implicated in growth factor-mediated angiogenesis (22, 23), we next investigated whether PI3K/p85 signaling mediates WISP-3-induced angiogenesis, CM was collected from MH7A cells pretreated with LY294002 (10 μM), Wortmannin (5 μM), or transfected with p85 siRNA prior to stimulation with recombinant WISP-3 (100 ng/mL). Representative images and quantitative analysis demonstrated that both pharmacological inhibition of PI3K and p85 knockdown significantly attenuated WISP-3-induced endothelial tubular formation (**Figures 3A, B**). Similarly, PDGF-B mRNA expression was markedly reduced following PI3K inhibition or p85 knockdown (**Figure 3C**). Representative Western blot and quantitative analysis further demonstrated that recombinant WISP-3 (100 ng/mL) rapidly induced p85 phosphorylation in a time-dependent manner (**Figures 3D, E**), whereas efficient p85 knockdown was confirmed by Western blot analysis (**Figures 3F, G**). Collectively, these findings demonstrate that activation of PI3K/p85 signaling is required for WISP-3-induced PDGF-B expression and angiogenic activity in MH7A cells.

### Akt activation links p85 signaling to PDGF-B upregulation in response to WISP-3

**Figure 4:**
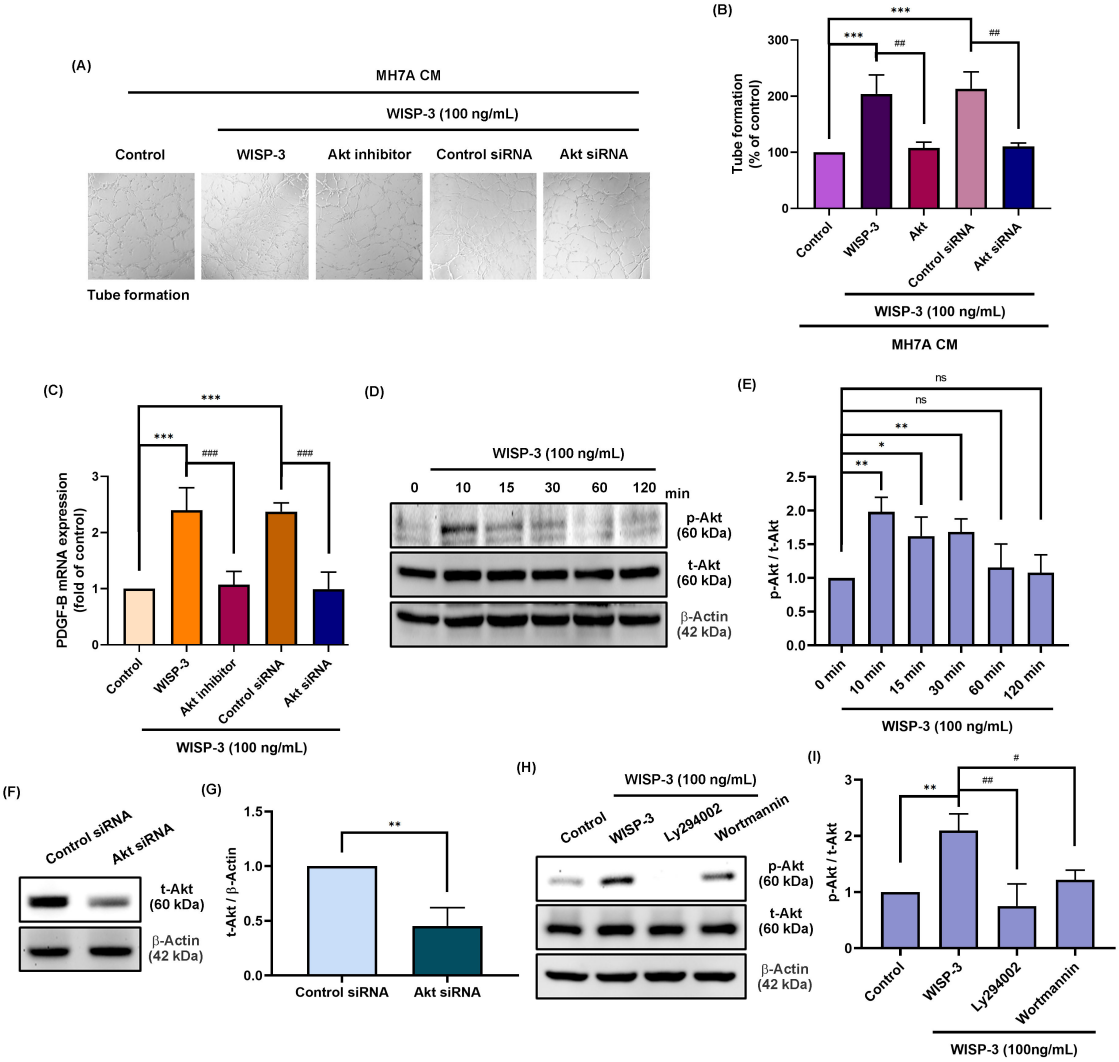
Akt mediates WISP-3-induced PDGF-B expression and angiogenesis. **(A, B)** Representative images and quantitative analysis of HUVEC tube formation using CM collected from MH7A cells pretreated with the Akt inhibitor (5 μM) or transfected with Akt siRNA prior to stimulation with recombinant WISP-3 (100 ng/mL). **(C)** PDGF-B mRNA expression in MH7A cells pretreated with the Akt inhibitor (5 μM) or transfected with Akt siRNA prior to stimulation with recombinant WISP-3 (100 ng/mL). **(D, E)** Representative Western blots and quantitative analysis of Akt phosphorylation following stimulation with recombinant WISP-3 (100 ng/mL) for the indicated time periods. **(F, G)** Representative Western blots and quantitative analysis confirming Akt knockdown efficiency. **(H, I)** Representative Western blots and quantitative analysis of Akt phosphorylation in MH7A cells pretreated with LY294002 (10 μM) or Wortmannin (5 μM) prior to stimulation with recombinant WISP-3 (100 ng/mL). Data are presented as the mean ± SD from at least three independent experiments. Statistical significance was analyzed using one-way ANOVA followed by Tukey’s *post hoc* test. *p < 0.05, **p < 0.01, ***p < 0.001 versus the control group; #*p* < 0.05, ##*p* < 0.01, ###*p* < 0.001 versus the WISP-3-treated group; ns, not significant.

Following the identification of PI3K/p85 as an upstream regulator, we next investigated whether Akt mediates WISP-3-induced signaling. Pharmacological inhibition or siRNA-mediated knockdown of Akt significantly impaired the ability of conditioned medium from WISP-3-treated MH7A cells to promote endothelial tube formation (**Figures 4A, B**). Consistent with these findings, inhibition of Akt also reduced PDGF-B mRNA expression (**Figure 4C**). WISP-3 rapidly induced Akt phosphorylation in a time-dependent manner, whereas Akt silencing effectively reduced Akt protein expression (**Figures 4D–G**). Furthermore, pretreatment with the PI3K inhibitors LY294002 or Wortmannin markedly suppressed WISP-3-induced Akt phosphorylation (**Figures 4H, I**), placing Akt downstream of PI3K/p85 activation. Together, these findings demonstrate that Akt serves as a critical downstream mediator linking PI3K/p85 signaling to PDGF-B upregulation and angiogenic activity induced by WISP-3.

### mTOR activation is required for WISP-3-induced PDGF-B expression downstream of p85/Akt

**Figure 5:**
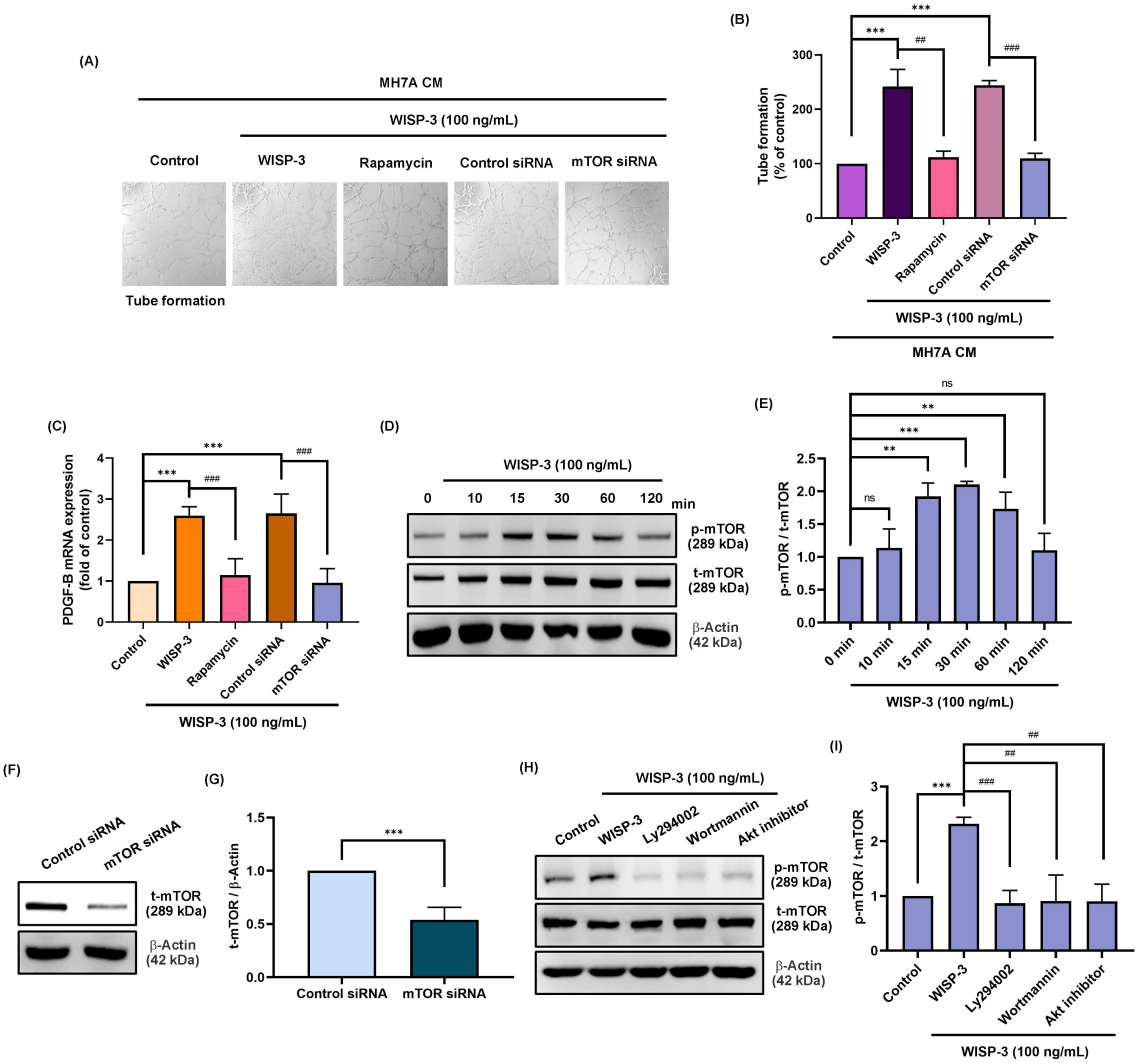
mTOR functions downstream of p85/Akt to mediate WISP-3-induced PDGF-B expression and angiogenesis. **(A, B)** Representative images and quantitative analysis of HUVEC tube formation using CM collected from MH7A cells pretreated with rapamycin (10 μM) or transfected with mTOR siRNA prior to stimulation with recombinant WISP-3 (100 ng/mL). **(C)** PDGF-B mRNA expression in MH7A cells pretreated with rapamycin (10 μM) or transfected with mTOR siRNA prior to stimulation with recombinant WISP-3 (100 ng/mL). **(D, E)** Representative Western blots and quantitative analysis of mTOR phosphorylation following stimulation with recombinant WISP-3 (100 ng/mL) for the indicated time periods. **(F, G)** Representative Western blots and quantitative analysis confirming mTOR knockdown efficiency. **(H, I)** Representative Western blots and quantitative analysis of mTOR phosphorylation in MH7A cells pretreated with LY294002 (10 μM), Wortmannin (5 μM), or the Akt inhibitor (5 μM) prior to stimulation with recombinant WISP-3 (100 ng/mL). Data are presented as the mean ± SD from at least three independent experiments. Statistical significance was analyzed using one-way ANOVA followed by Tukey’s *post hoc* test. *p < 0.05, **p < 0.01, ***p < 0.001 versus the control group; #*p* < 0.05, ##*p* < 0.01, ###*p* < 0.001 versus the WISP-3-treated group; ns, not significant.

We next determined whether mTOR functions downstream of PI3K/Akt during WISP-3 signaling. Treatment with rapamycin or siRNA-mediated knockdown of mTOR significantly diminished endothelial tube formation induced by conditioned medium from WISP-3-stimulated MH7A cells (**Figures 5A, B**). Suppression of mTOR also markedly reduced PDGF-B mRNA expression (**Figure 5C**). Time-course analysis showed that WISP-3 stimulated rapid phosphorylation of mTOR, while efficient mTOR knockdown was confirmed by Western blotting (**Figures 5D–G**). In addition, inhibition of upstream PI3K or Akt signaling attenuated WISP-3-induced mTOR phosphorylation (**Figures 5H, I**), confirming that mTOR activation depends on PI3K/Akt signaling. These results identify mTOR as a downstream effector of the PI3K/Akt pathway that is required for WISP-3-induced PDGF-B expression and angiogenic activity.

### WISP-3 regulates PDGF-B expression through suppression of hsa-let-7b-5p in MH7A cells

**Figure 6:**
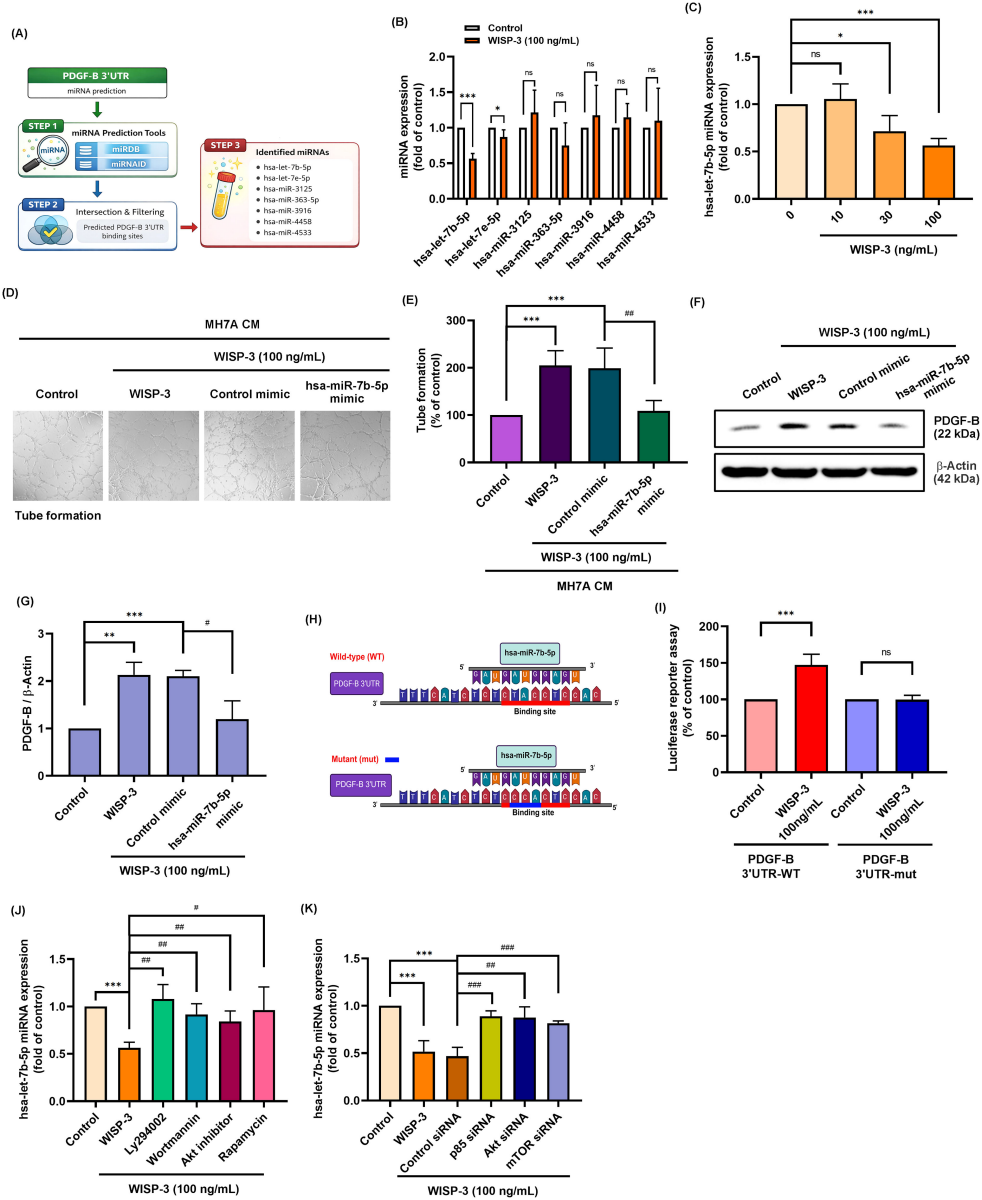
Hsa-let-7b-5p mediates WISP-3-induced PDGF-B expression and angiogenesis. **(A)** Workflow illustrating the identification of candidate miRNAs targeting the PDGF-B 3′UTR using miRNA prediction databases. **(B)** Expression of candidate miRNAs in MH7A cells following stimulation with recombinant WISP-3 (100 ng/mL). **(C)** Expression of hsa-let-7b-5p in MH7A cells treated with increasing concentrations of recombinant WISP-3 (0-100 ng/mL). **(D, E)** Representative images and quantitative analysis of HUVEC tube formation using CM collected from MH7A cells transfected with a control mimic or hsa-let-7b-5p mimic prior to stimulation with recombinant WISP-3 (100 ng/mL). **(F, G)** Representative Western blot and quantitative analysis of PDGF-B protein expression in MH7A cells transfected with a control mimic or hsa-let-7b-5p mimic prior to stimulation with recombinant WISP-3 (100 ng/mL). **(H, I)** Schematic illustration of the wild-type and mutant PDGF-B 3′UTR luciferase reporter constructs and the corresponding luciferase reporter assay. **(J, K)** Expression of hsa-let-7b-5p in MH7A cells following pharmacological inhibition or siRNA-mediated knockdown of the PI3K/Akt/mTOR signaling pathway prior to stimulation with recombinant WISP-3 (100 ng/mL). Data are presented as the mean ± SD from at least three independent experiments. Statistical significance was analyzed using one-way ANOVA followed by Tukey’s *post hoc* test. *p < 0.05, **p < 0.01, ***p < 0.001 versus the control group; #*p* < 0.05, ##*p* < 0.01, ###*p* < 0.001 versus the WISP-3-treated group; ns, not significant.

**Figure 7:**
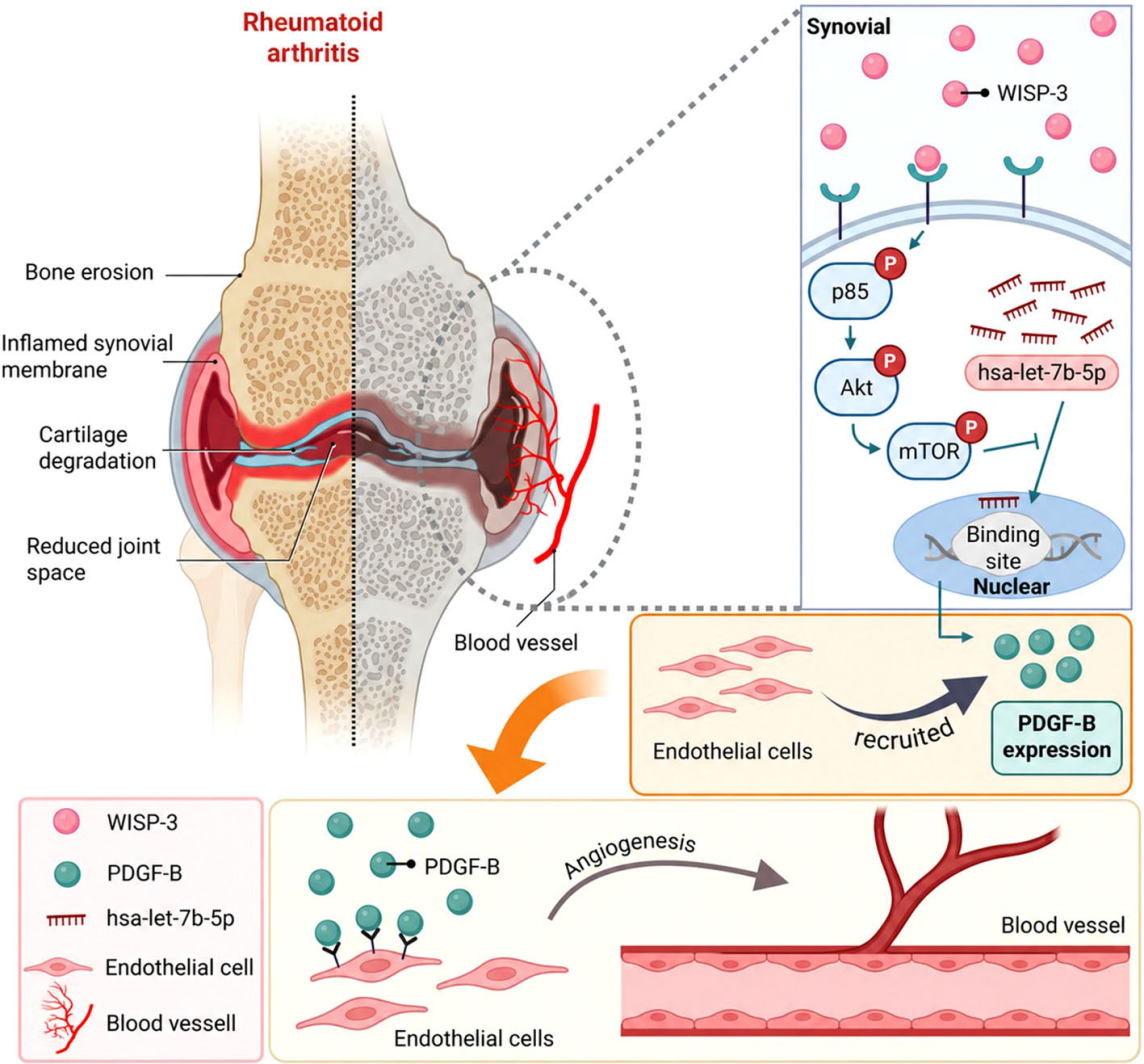
Proposed mechanism of WISP-3-mediated angiogenesis in RA. Schematic illustration of the proposed signaling mechanism. WISP-3 activates the p85/Akt/mTOR signaling cascade, resulting in suppression of hsa-let-7b-5p, and increased PDGF-B expression, and enhanced angiogenesis.

To investigate the post-transcriptional mechanism underlying WISP-3-induced PDGF-B expression, candidate miRNAs targeting the PDGF-B 3′UTR were identified using two independent miRNA prediction databases (**Figure 6A**). Among the predicted candidates, hsa-let-7b-5p was significantly downregulated following WISP-3 stimulation (**Figure 6B**). Moreover, WISP-3 suppressed hsa-let-7b-5p expression in a dose-dependent manner (**Figure 6C**). To determine whether hsa-let-7b-5p contributes to WISP-3-induced angiogenesis, MH7A cells were transfected with a hsa-let-7b-5p mimic prior to WISP-3 stimulation. Transfection with the hsa-let-7b-5p mimic significantly attenuated WISP-3-induced endothelial tube formation and PDGF-B protein expression, as demonstrated by representative images, quantitative analyses, and Western blot analysis (**Figures 6D–G**). To further validate the interaction between hsa-let-7b-5p and PDGF-B, wild-type and mutant PDGF-B 3′UTR luciferase reporter constructs were generated (**Figure 6H**). Luciferase reporter analysis demonstrated that WISP-3 significantly increased luciferase activity of the wild-type PDGF-B 3′UTR reporter, whereas mutation of the predicted hsa-let-7b-5p binding site abolished this response (**Figure 6I**), supporting that the predicted binding site is required for WISP-3-mediated regulation of PDGF-B. Finally, both pharmacological inhibition and siRNA-mediated knockdown of the PI3K/Akt/mTOR signaling components significantly restored hsa-let-7b-5p expression suppressed by WISP-3 (**Figures 6J, K**), indicating that hsa-let-7b-5p acts downstream of PI3K/Akt/mTOR signaling. Collectively, these findings indicate that WISP-3 suppresses hsa-let-7b-5p through activation of the PI3K/Akt/mTOR signaling pathway, thereby promoting PDGF-B expression and angiogenic activity in MH7A cells (**Figure 7**).

## Discussion

Angiogenesis is a hallmark of RA and plays a pivotal role in synovial hyperplasia, pannus formation, and progressive joint destruction (24). Although numerous angiogenic mediators have been implicated in RA, the upstream molecular events coordinating these responses remain incompletely understood. In the present study, we identified WISP-3 as a previously unrecognized regulator of RA-associated angiogenesis and demonstrated that it promotes PDGF-B expression through activation of the p85/Akt/mTOR signaling pathway and suppression of hsa-let-7b-5p. Notably, among all CCN family members analyzed, WISP-3 exhibited the most consistent increase across two independent GEO datasets and clinical samples, supporting its selection for subsequent mechanistic investigation. Although WISP-3 has previously been associated with progressive pseudorheumatoid dysplasia (PPRD) caused by loss-of-function mutations, its contribution to inflammatory joint diseases has remained largely unexplored. Our findings therefore expand the biological role of WISP-3 beyond skeletal development and identify it as a potential regulator of pathological angiogenesis in RA.

Interestingly, previous studies have demonstrated that WISP-3 promotes VEGF-A expression in chondrosarcoma cells and enhances VEGF-C-dependent lymphangiogenesis through miRNA-dependent mechanisms (17, 18). In contrast, our transcriptomic screening identified PDGF-B rather than VEGF-A as the major angiogenic factor regulated by WISP-3 in RA synovial fibroblasts. Consistent with this observation, analysis of the GSE12021 dataset showed no obvious increase in VEGF-A expression in RA synovial tissues compared with normal or osteoarthritic tissues. These findings suggest that the downstream angiogenic programs regulated by WISP-3 are highly dependent on cellular context and disease microenvironment. Whereas VEGF-A may represent the predominant downstream effector in chondrosarcoma, PDGF-B appears to play a more prominent role in RA synovial fibroblasts. This context-dependent regulation is consistent with the diverse biological functions of CCN family proteins and highlights the complexity of WISP-3 signaling across different pathological conditions.

Mechanistically, our results demonstrate that WISP-3 activates the p85/Akt/mTOR signaling cascade to regulate PDGF-B expression. Pharmacological inhibition and siRNA-mediated knockdown of p85, Akt, and mTOR attenuated WISP-3-induced PDGF-B expression and angiogenic activity, supporting a central role for this pathway. The p85/Akt/mTOR pathway has been widely implicated in cell survival, metabolism, angiogenesis, and inflammatory responses, and its activation has previously been documented in RA synovial tissues (25, 26). Pharmacological inhibition and gene silencing consistently attenuated WISP-3-induced PDGF-B expression and endothelial tube formation, indicating that this signaling axis is indispensable for the angiogenic effects of WISP-3. Rather than functioning as an isolated signaling molecule, WISP-3 appears to act upstream of a canonical angiogenic pathway that integrates extracellular stimulation with intracellular transcriptional responses. These findings provide a mechanistic framework linking extracellular matrix-associated proteins to pathological angiogenesis in RA.

In addition to intracellular signaling, our study demonstrates that WISP-3 regulates PDGF-B expression through post-transcriptional control of hsa-let-7b-5p. Members of the let-7 family participate in immune regulation, inflammation, and vascular remodeling, and altered let-7 expression has been reported in patients with RA (14). Here, we identified hsa-let-7b-5p as a downstream target of WISP-3 and demonstrated that restoration of hsa-let-7b-5p expression markedly suppressed PDGF-B expression and angiogenic activity. Furthermore, luciferase reporter assays confirmed direct interaction between hsa-let-7b-5p and the PDGF-B 3′UTR, supporting PDGF-B as a direct downstream target of hsa-let-7b-5p. These findings reveal a previously unrecognized WISP-3-hsa-let-7b-5p-PDGF-B regulatory axis and further emphasize the importance of coordinated signaling and microRNA-mediated regulation during pathological angiogenesis.

Several limitations of this study should be acknowledged. First, the functional analyses were primarily performed using *in vitro* models of synovial fibroblasts and endothelial cells. Although these systems are widely used to investigate RA-associated angiogenesis, validation in animal models will be necessary to determine the contribution of WISP-3 to synovial vascular remodeling within the complex joint microenvironment. Second, although angiogenesis is closely linked to immune cell infiltration during RA progression, the present study focused primarily on endothelial responses and did not directly investigate the effects of WISP-3-induced conditioned medium on immune cell recruitment or polarization. Future studies examining macrophage chemotaxis, macrophage polarization, and T-cell responses will be important for defining the immunological significance of the WISP-3/PDGF-B signaling axis and further extending its relevance to the immune microenvironment of RA. Finally, although our data establish WISP-3 as an upstream regulator of PDGF-B, the molecular mechanisms responsible for WISP-3 upregulation in RA remain unknown and warrant further investigation.

In summary, this study identifies WISP-3 as a previously unrecognized regulator of pathological angiogenesis in RA. Through activation of the p85/Akt/mTOR signaling pathway and suppression of hsa-let-7b-5p, WISP-3 enhances PDGF-B expression and promotes angiogenic responses in synovial fibroblasts. These findings provide new mechanistic insight into synovial vascular remodeling and establish the WISP-3-p85/Akt/mTOR-let-7b-5p-PDGF-B signaling axis as a potential therapeutic target for pathological angiogenesis in rheumatoid arthritis.

## Data Availability

The raw data supporting the conclusions of this article will be made available by the authors, without undue reservation.
