# Supplementary material for: WISP-3 promotes PDGF-B-mediated angiogenesis through suppression of let-7b-5p in rheumatoid arthritis
Source: Front Immunol. 2026 Jul 29;17:1842824. doi: 10.3389/fimmu.2026.1842824 (PMC13462055; doi:10.3389/fimmu.2026.1842824)
Supplement: Supplementary file 1 [file Table1.doc]

**Table 1.**

List of PCR primer used for the experiments

| **Target mRNA** | **Forward primer (5’→3’)** | **Reverse primer (5’→3’)** |
| --- | --- | --- |
| **VEGF-A** | AGGGCAGAATCATCACGAAGT | AGGGTCTCGATTGGATGGCA |
| **VEGF-C** | GGCTGGCAACATAACAGAGAA | CCCCACATCTATACACACCTCC |
| **VEGF-D** | CCTGAAG AAGATCGCTGTTC | GAGAGCTGGTTCCTGGAGAT |
| **PDGF-A** | GCAAGACCAGGACGGTCATTT | GGCACTTGACACTGCTCGT |
| **PDGF-B** | CTCGATCCGCTCCTTTGATGA | CGTTGGTGCGGTCTATGAG |
| **PDGF-C** | ATTTGGGCTTGAAGACCCAGA | CCAGCGCCCTAATATAGTTCCA |
| **PDGF-D** | ACGGATACAGCTAGTGTTTGACA | GTCCACACCATCGTCCTCTAATA |
| **ANGPT1** | AGCGCCGAAGTCCAGAAAAC | TACTCTCACGACAGTTGCCAT |
| **ANGPT2** | AACTTTCGGAAGAGCATGGAC | CGAGTCATCGTATTCGAGCGG |
| **GAPDH** | ACCACAGTCCATGCCATCAC | TCCACCACCCTGTTGCTGTA |
|  | | |
| **Target miRNA** | **Forward primer (5’→3’)** | |
| **has-let-7b-5p** | TGAGGTAGTAGGTTGTGTGGTT | |
| **has- let-7e-5p** | TGAGGTAGGAGGTTGTATAGTT | |
| **has-miR-3125** | TAGAGGAAGCTGTGGAGAGA | |
| **has-miR-363-5p** | CGGGTGGATCACGATGCAATTT | |
| **has-miR-3916** | AAGAGGAAGAAATGGCTGGTTCTCAG | |
| **has-miR-4458** | AGAGGTAGGTGTGGAAGAA | |
| **has-miR-4533** | TGGAAGGAGGTTGCCGGACGCT | |
